# Axonal Transport, Phase-Separated Compartments, and Neuron Mechanics - A New Approach to Investigate Neurodegenerative Diseases

**DOI:** 10.3389/fncel.2018.00358

**Published:** 2018-10-09

**Authors:** Martin Nötzel, Gonzalo Rosso, Stephanie Möllmert, Anne Seifert, Raimund Schlüßler, Kyoohyun Kim, Andreas Hermann, Jochen Guck

**Affiliations:** ^1^Biotechnology Center, Dresden University of Technology, Dresden, Germany; ^2^Department of Neurology, Technische Universität Dresden, Dresden, Germany; ^3^Center for Regenerative Therapies (CRTD), Technische Universität Dresden, Dresden, Germany; ^4^German Center for Neurodegenerative Diseases, Dresden, Germany

**Keywords:** neurodegenerative disease, amyotrophic lateral sclerosis, cell mechanics, phase separation, stress granules, atomic force microscopy, Brillouin microscopy, optical diffraction tomography

## Abstract

Many molecular and cellular pathogenic mechanisms of neurodegenerative diseases have been revealed. However, it is unclear what role a putatively impaired neuronal transport with respect to altered mechanical properties of neurons play in the initiation and progression of such diseases. The biochemical aspects of intracellular axonal transport, which is important for molecular movements through the cytoplasm, e.g., mitochondrial movement, has already been studied. Interestingly, transport deficiencies are associated with the emergence of the affliction and potentially linked to disease transmission. Transport along the axon depends on the normal function of the neuronal cytoskeleton, which is also a major contributor to neuronal mechanical properties. By contrast, little attention has been paid to the mechanical properties of neurons and axons impaired by neurodegeneration, and of membraneless, phase-separated organelles such as stress granules (SGs) within neurons. Mechanical changes may indicate cytoskeleton reorganization and function, and thus give information about the transport and other system impairment. Nowadays, several techniques to investigate cellular mechanical properties are available. In this review, we discuss how select biophysical methods to probe material properties could contribute to the general understanding of mechanisms underlying neurodegenerative diseases.

## Introduction

**FIGURE 1:**
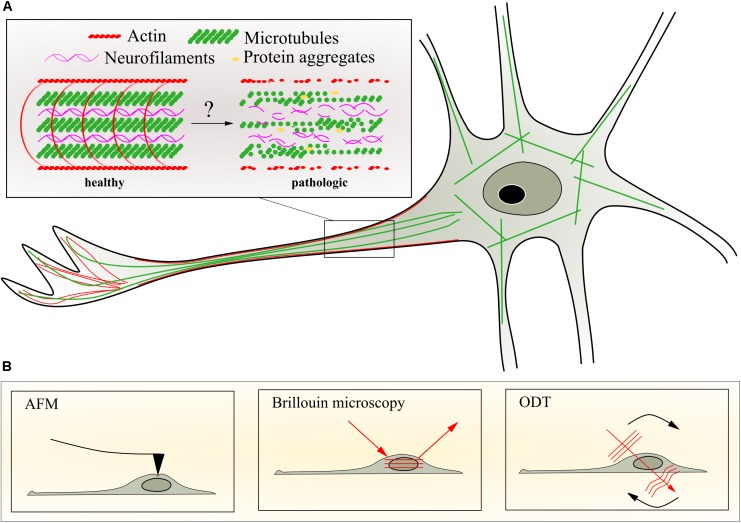
Neuronal cytoskeleton and selected methods to quantify mechanical properties. **(A)** Schematic representation of potential changes of axonal cytoskeleton organization during progression of motor neuron diseases caused by transport deficiencies, microtubule depolymerization, and aggregation of intermediate filaments or impaired actin dynamics. **(B)** Schematic representation of techniques to assess biophysical changes in the living neuron such as atomic force microscopy (AFM), Brillouin microscopy, and optical diffraction tomography (ODT). For details of the different techniques, see text.

Neurons contain three different types of cytoskeletal filaments: microtubules (MTs), actin filaments, and neurofilaments (NFs) (**Figure 1A**). These cytoskeletal components fulfill important physiological functions during nervous system development and maturation and account for the neuronal structural organization (Franze et al., 2013). For instance, the neuronal cytoskeleton is involved in migration, pathfinding (Bearce et al., 2015), and axonal transport (Maday et al., 2014). The organization of the cytoskeleton, in particular the MT network, determines axonal transport dynamics (Maday et al., 2014). Axonal transport is crucial for neuronal function and strictly depends on an active transport machinery able to move cargoes including but not limited to organelles such as mitochondria, synaptic vesicles, proteins, and RNA along the MT network. Biosynthesis of molecules in the neuron’s soma and their transport across long axons (up to 1 m in adult humans) is pivotal for the physiology and survival of neurons. The active axonal transport of molecules along the MTs is carried out by the molecular motors kinesin and dynein, thereby providing anterograde and retrograde transport, respectively. Also, the actin cytoskeleton is involved in transport, but on shorter length scales compared to MTs (Rogers and Gelfand, 1998).

The cytoskeleton is also chiefly important for determining the mechanical properties of neurons and their axons. This link between physiological transport and pathological processes on the one hand, and these mechanical properties on the other, can be exploited to acquire additional and new information using appropriate measurement techniques. Advanced biophysical methods to investigate cellular mechanics such as atomic force microscopy (AFM) (**Figure 1**), traction force microscopy, and micropillar arrays have significantly contributed to the understanding of cytoskeletal mechanics, forces acting on and generated by isolated adherent cells *in vitro*, as well as their intrinsic material properties (Rodriguez et al., 2013). These methods quantify cell mechanical properties such as elasticity and viscosity, surface tension, or traction forces. These properties differ for distinct cell types and can, in combination with various microscopy techniques, be attributed to certain cellular compartments and cellular functions (Lammerding, 2011; Haase et al., 2015).

Neurodegenerative diseases such as amyotrophic lateral sclerosis (ALS), Parkinson’s disease (PD), or frontotemporal dementia (FTD) are serious afflictions and can cause a dramatic decrease in quality of life and lifetime. Such diseases lead to impaired neurological structure and function with manifold neurological symptoms. A well-known common feature is the accumulation of toxic aggregates within the neurons (Taylor, 2002). Also, transport processes, which are tightly bound to the structure and function of the cytoskeleton, are altered (De Vos et al., 2008). For example, cytoskeletal components have been found to accumulate in neurons of ALS patients. These components can be NFs within the cytoplasm (Julien and Beaulieu, 2000), as well as spheroids composed of NFs and peripherin (an NF-associated protein; Chadwick and Goode, 2006). In some cases, axonal transport processes are disrupted as a direct consequence. However, more common are pathological aggregates that include RNA-binding proteins (RBPs) and form when a physiological protective measure goes wrong, either through prolonged stress or disease-associated mutations.

In this article, we describe AFM, Brillouin microscopy (BM), and optical diffraction tomography (ODT) as current biophysical techniques utilized to investigate neuronal mechanics. We discuss how they can be applied to study the link between the cytoskeleton and axonal transport-deficient neurons, as well as to quantify pathological aggregates in neurological diseases.

## Cytoskeletal Mechanics and Axonal Transport

Filamentous actin (F-actin) is a helical ubiquitous protein which consists of globular actin (G-actin) subunits. Actin filaments are highly dynamic and the polymerization or depolymerization of G-actin molecules depends on the G-actin concentration. Actin dynamics is highly regulated *in vivo*. Proteins such as profilin, filamin, and the Arp2/3 complex regulate the process of assembly and disassembly which is controlled by enzymes and G-proteins. The functions of actin can be manifold, including mechanical stabilization of the cell and transport processes (Kuznetsov et al., 1992). Especially for complex shaped structures such as neurons, the actin network is not homogeneously distributed through the cell but can form several structures as reviewed by Leterrier et al. (2017) (**Figure 1A**). The density of F-actin differs throughout the neuron; for instance, it is particularly high in the growth cone. Such local variations in the actin network can also modify local mechanical properties.

Actin filaments can be described as semi-flexible polymers (MacKintosh et al., 1995). This means there is a certain filament length, called the persistence length, over which they do not bend much due to thermal fluctuation. The persistence length for actin is about 15 μm (Howard and Clark, 2002). The elastic modulus has been measured to be 1.8 GPa using *in vitro* nanomanipulation with microneedles (Kojima et al., 1994). The filaments can form actin networks when they are linked by proteins. Those proteins are called crosslinkers and the final actin structure depends on their size, binding properties, and concentration (Gardel et al., 2008). Such networks can be described using different models, which all have in common that the structure can be characterized by mesh size, elastic modulus, persistence length, and the characteristic length of the network or the distance between cross-links for cross-linked networks (MacKintosh et al., 1995; Pujol et al., 2012). F-actin and crosslinkers can be mixed *in vitro* to form a homogenously crosslinked actin network to be used in deformation experiments. In such investigations, the response to small stresses is generally viscoelastic. Details depend on the experimental timescale or the frequency, the applied stress and again the model (Stricker et al., 2010; Wu et al., 2018). Furthermore, experiments have been done *in vitro* on actin network models which are able to actively change and therefore mimic the in-cell behavior (Gardel et al., 2008; Fletcher and Geissler, 2009). These models are often based on the well-studied bacterial pathogen *Listeria monocytogenes* (Tilney and Portnoy, 1989). The elastic modulus determined by AFM-based microrheology for dendritic actin networks is about 1 kPa (Chaudhuri et al., 2007). By comparison, the elastic modulus of an axon has been measured to be about 9.5 kPa using AFM indentation measurements (Ouyang et al., 2013). Of note, an earlier study, using a less pointed, spherical AFM indenter reported values much closer to the situation *in vitro* (Lu et al., 2006). The discrepancy can probably be explained by the different levels of stress applied with the two different probes (Wu et al., 2018). It has been shown that actin can also show mechanosensing behavior (Hayakawa et al., 2012). This has to be taken into account for measurement methods which directly interact with the cell such as AFM.

The MT network (**Figure 1A**) contributes to the maintenance of the cell structure and is almost entirely responsible for the physiologically crucial axonal transport in neurons. Due to their contribution to maintaining the cell shape, MTs are permanently exposed to internal and external forces such as shown by the curvature of MT filaments in living cells (Odde et al., 1999; Brangwynne et al., 2006). Gittes and colleagues studied *in vitro* the flexural MT rigidity using thermal fluctuations to bend filaments in solution. They showed that MTs are rigid filaments and can withstand large deformation forces (Gittes et al., 1993). Other approaches to study the flexural MT rigidity include optical traps and beads (Felgner et al., 1996; Kikumoto et al., 2006) as well as bending of filaments *via* hydrodynamic flow (Gittes et al., 1993; Hawkins et al., 2010). Although the obtained results differ among implemented methods (Kasas and Dietler, 2007), the accumulated data indicate that MT filaments are stiff filamentous proteins and show significantly higher stiffness values compared to actin filaments (Gittes et al., 1993; Hawkins et al., 2010). Interestingly, optical tweezers (Felgner et al., 1997). Mechanical probing of isolated MTs utilizing AFM (Schaap et al., 2007) have shown that neuronal MTs can modify their stability increasing their resistance to rupture, which depends on their association with microtubule-associated-proteins (MAPs) such as tau. Furthermore, phosphorylation of MAPs has been shown to affect MT stability and therefore axonal transport (Dixit et al., 2008). In addition, mutations in tau might be associated with axonal transport deficiencies and were found in patients with neurological disorders such as FTD and atypical Parkinson syndromes (Hutton et al., 1998). Another mechanism that might contribute to the mechanical properties of neuronal cells might include MT post-translational modifications such as tubulin detyrosination, A2-tubulin generation, polyglutamylation, and acetylation among others (Janke and Bulinski, 2011; Song et al., 2013). It has been suggested that MT acetylation is required for MT mechanical stabilization *in vivo* and *in vitro*, whereas depletion of the enzyme acetyltransferase significantly increase MT breakage in cells (Xu et al., 2017).

In the cell mechanics field, the contribution of the MT network to the overall cell elasticity has been studied in several non-neuronal cell types utilizing different biophysical tools (Rotsch and Radmacher, 2000; Hoffman et al., 2006; Mietke et al., 2015). Kubitschke et al. (2017) have shown that the actin and MT networks contribute differently to the cell elasticity when exposed to different types of strains. Due to their long axons and the need for axonal transport machinery, which relies on MT stability and associated proteins, neuronal cells are excellent candidates to investigate the connection between mechanical properties of MTs and axonal transport *in vivo* (Tang-Schomer et al., 2010; Magdesian et al., 2012).

The intermediate filament (IF) network (**Figure 1A**) provides the cell with remarkable stability. Different biophysical approaches have been utilized to investigate IF mechanical properties *in vitro* (Köster et al., 2015; Scekic-Zahirovic et al., 2016). For instance, using AFM-based mechanical measurements to stretch various types of isolated IFs, Kreplak et al. (2005) showed that IFs can stretch up to 3.6-fold before they rupture, and suggested that NFs, the specific IFs found in nerve cells, may act as mechanical shock absorbers in living cells. In addition, other types of IFs such as desmin and keratin show similar stretching behaviors when compared to NFs (Kreplak et al., 2005). Neurons express different types of IFs proteins depending on the developmental stage or the localization in the nervous system (Toivola et al., 2005). NFs are abundant in the mature nervous system along large myelinated axons where their main function is the maintenance of axon caliber (Perrot et al., 2008; Yuan et al., 2012). Inside the axons, NFs are organized in heteropolymers composed of four subunits called heavy (NFH), medium (NFM), light (NFL), and α-internexin (Yuan et al., 2012). NFs have become of recent interest in clinical neuroscience since increased concentrations are found in different body fluids under diverse disease conditions (Khalil et al., 2018).

The three cytoskeletal filaments together form a structurally coupled network which contributes to the mechanical resilience that is important in cellular processes such as structural integrity, growth, and transport. The heterogeneous composition of the cytoskeleton in eukaryotic cells results in interesting emergent mechanical properties that cannot be explained by the simple mechanical contribution of its isolated components. There are evidences showing direct physical interaction between actin filaments, MTs, and NFs both *in vivo* and *in vitro* (Huber et al., 2015). For instance, Esue et al. (2006) have shown that actin and the IF vimentin directly interact *via* a tail domain in the vimentin molecule, and that cross-link may contribute to the mechanical stability of cells. Similar molecular interactions were found between NFs and MTs. Hisanaga and Hirokawa (1990) reported that dephosphorylation of NFs plays a role in the interaction between MTs and the NFH subunit. The latter might suggest that the cytoskeleton cross-talk and its intrinsic dynamic regulation mechanisms *via* cross-linking proteins could ultimately affect the mechanical properties of neurons.

Axonal transport relies predominantly on MTs. But especially for short distance, movement of mitochondria also actin plays an important role (Rogers and Gelfand, 1998). Osmotic pressure and motor proteins mediate the transport by generating endogenous forces (Guo et al., 2018). Without doubt, the cytoskeleton has the biggest influence on the mechanics, but other components of the cell contribute as well. These can be physiological (e.g., organelles) or pathological such as protein aggregates as they occur in many neurodegenerative diseases. Although the *in vitro* experiments described have significantly contributed to the understanding of the cytoskeleton and its mechanical properties, a cell is still much more complex. Especially polarized cells such as neurons are highly dynamic cellular machines which constantly experience and induce mechanical changes. The complex interaction of regulation processes involving a large number of proteins inside the cell can currently only be investigated utilizing *in vivo* models. This is particularly the case for diseased neurons.

While most studies on neurodegeneration so far have focused on the biochemical signals that lead to axon degeneration, little is known about the mechanical properties of diseased neurons and how the alterations in the axonal transport machinery and cytoskeletal architecture affect their mechanical resistance. There is strong evidence linking transport deficiencies and the emergence of ALS (Kreiter et al., 2018; Naumann et al., 2018). These changes are associated with several mutations in genes encoding proteins for MTs (Smith et al., 2014), actin (Wu et al., 2012), and NFs (Al-Chalabi et al., 1999). For instance, phosphorylation of MAPs has been shown to affect MT stability and therefore axonal transport (Dixit et al., 2008). Also, PD is associated with aberrant MT function (Pellegrini et al., 2017). In addition, mutations in tau, which regulates MT assembly, are associated with axonal transport deficiencies and were found in patients with neurological disorders, including FTD (Hutton et al., 1998; Wolfe, 2009) Alterations in the structural organization and transport of NFs in CNS neurons have been associated with many neurological disorders including ALS (Al-Chalabi et al., 1999; Cairns et al., 2004; Omary, 2004). Furthermore, a well-investigated pathological hallmark for several human neurodegenerative diseases including ALS, is the accumulation of hyper-phosphorylated NFs in the proximal axon of large motor neurons as an impairment in the axonal transport machinery (Manetto et al., 1988; Liu, 2011; De Vos and Hafezparast, 2017). The contribution of axonal cytoskeletal components to the overall elasticity of neurons has previously been investigated. Ouyang and colleagues carried out AFM nano-indentation measurements on isolated axons after treatment with several cytoskeleton destabilizing agents to test the contribution of these components to axon elasticity. It has been shown that the major contributors of axonal elasticity are the MTs, followed by NFs and F-actin (Ouyang et al., 2013). Multiple studies have shown that cytoskeleton disruption using cytochalasin D, nocodazole, paclitaxel, and other substances strongly affect the mechanical properties of various cell types (Reynolds et al., 2014; Golfier et al., 2017; Kanda and Gu, 2017). In neurons, the disruption of MTs significantly affects the cell mechanical properties (Ouyang et al., 2013) as well as axonal transport, e.g., slow component-b cargo transport (Roy et al., 2008).

In light of the aforementioned aspects, there is likely a connection between transport and cell mechanics which are linked *via* the cytoskeletal network. For example, the mechanical properties of central and peripheral healthy neurons and their ability to withstand compression while monitoring axonal transport has been investigated by Magdesian et al. (2012). They found pressure limits from which the axonal transport cannot recover without axonal damage. Loverde et al. (2011) has shown that mitochondrial transport diminishes as the axon is stretched. The combination of stretch and paclitaxel treatment has an especially strong effect on the axonal transport (Bober et al., 2015). Any study that investigates changes in the mechanical properties of degenerating neurons will likely also uncover mechanisms related to altered transport and can, thus, help to better understand the progression, causes, and consequences of the disease.

## Phase Separation and Aggregate Formation

Apart from cytoskeletal deficiencies linked to transport, there is another, more recently discovered phenomenon associated with many neurodegenerative diseases including PD, Alzheimer’s disease (AD), Huntington’s disease (HD), ALS, and FTD (Sin and Nollen, 2015), which also has a mechanical component. RBPs contain a low sequence complexity, prion-like domain with a high content of glycine, which accounts for their tendency to aggregate (Han et al., 2012). Studies have shown that ALS-associated mutations of TDP-43 largely occur within this low-complexity domain, thereby increasing the tendency to aggregate even further (Wolozin and Collection, 2014). Under physiological conditions, RBPs can accumulate together with mRNA, the 40S ribosomal subunit, and other proteins into membraneless compartments called stress granules (SGs) (Dewey et al., 2012; Mateju et al., 2017). This occurs, for example, in order to halt the translation of specified proteins under stress conditions and focus on the production of protective proteins necessary for cell survival. This is in line with the finding that SGs form when translation is at the initiation step. For this purpose, eukaryotic elongation factor 2 alpha is phosphorylated, which prevents assembly of the ternary complex (eIF2α-GTP-tRNAMet). It then no longer binds to the 48S pre-initiation complex, and translation is stalled (Dewey et al., 2012).

This process of aggregation can create large macromolecular complexes of RBPs. However, if mutated RBPs are integrated into these structures, they can undergo a pathological low-complexity domain-driven liquid-to-solid phase transition, thereby resulting in solidified SGs (Bowden and Dormann, 2016). These solidified granules have lost their dynamic properties and hence the ability to fulfill their physiological functions. This may result in impaired stress response and mRNA transport, altered local translation, and the formation of pathological aggregates, all of which may contribute to neuron dysfunction and ultimately neurodegeneration (Bowden and Dormann, 2016). The duration of stress, and possibly other factors such as the efficiency of the protein quality control system, greatly changes the composition of SGs in time and space (Mateju et al., 2017). SGs also gain size by MT instability (Chernov et al., 2009; Dewey et al., 2012). Additionally, the formation of SGs is largely dependent on the concentration of a respective protein. There is a sensitive equilibrium of molecules localized in the cytoplasm and in the nucleus, and anything that shifts this equilibrium will also shift the amount of aggregation (Wolozin and Collection, 2014). This is best described in the FUS-ALS pathology, where mutated FUS is mislocalized out of the nucleus into the cytoplasm, where it accumulates and colocalizes with SG markers (Wolozin and Collection, 2014). While proteins with ALS-associated mutations, such as TDP-43 and FUS, are non-essential for the formation of SGs, they are closely associated with these pathological aggregates (Dewey et al., 2012).

The physical properties of SGs have also been studied *in vitro*. It has been shown that wild-type recombinant RBPs demix from an aqueous solution and form liquid-like droplets. The intrinsically disordered prion-like domains of RBPs have been found to be sufficient for this so-called liquid-liquid phase separation. These two phases then coexist stably, while one is enriched for RNAs and RBPs, forming a compartment, which allows diffusion of molecules within, but is separated from the surrounding milieu by a free energy potential barrier. This is an essential process for the formation of multiple membraneless organelles involved for instance in RNA metabolism (Boeynaems et al., 2017). Another example is the formation of P bodies, where its components have a higher affinity with each other than they do with respect to cytoplasmic molecules. This inequality in affinities is what drives the phase separation and distinction of the P body from the cytoplasm (Hyman et al., 2014).

Over time, and especially during disease, these liquid-like droplets can mature to more fibrillary states, a process which is accelerated by proteins with disease causing mutations like those found in neurological disorders (Boeynaems et al., 2017). Specifically, the arginine-rich dipeptide repeats in C9orf72 are associated with ALS and FTD (Boeynaems et al., 2017). Larger SGs will attract even more misfolded proteins, which further adds to the transformation into solid compartment (Mateju et al., 2017). In addition, misfolded proteins expose their aggregation prone domains to the cellular environment – domains that would otherwise be structurally concealed – enhancing their cumulative properties (Sin and Nollen, 2015). Consequently, pathological aggregates may either form directly through the accumulation of mutated protein, potentially including RNA and RBPs, or from SGs which undergo a pathological liquid-to-solid phase transition.

The formation of pathological aggregates is however not solely regulated by its included proteins. HD is characterized by the neuronal accumulation of mutant Huntingtin (mHtt), which is a polyglutamine (polyQ) protein. One important regulator specific for this protein class is the C-terminal Hsp70 (heat shock protein 70)-interacting protein (CHIP), which has been identified to mediate the solubility of mutant polyQ proteins through its interaction with chaperones (Miller, 2005). By contrast, the ubiquitin conjugating enzyme Ube2W has been found to have a negative effect on aggregate formation and disease progression. In cultured cells with deficient Ube2W activity, decreased mHtt aggregate formation and increased levels of soluble monomers has been observed (Wang et al., 2018).

As SGs increase in size during their transition to form pathological aggregates, they may sterically impair cellular processes such as cytoskeletal assembly or cellular transport, as do other cellular components when they increase in size. This is in line with the finding that induced mitochondrial swelling reduces organelle trafficking of mitochondria and lysosomes in rat primary neurons (Kaasik et al., 2007). For lysosomes, the degree of impairment was dependent on the neurite diameter: the wider the neurite, the weaker the effect on lysosomal trafficking. Interestingly, both transport defects were ATP independent, as chemical modifiers affecting mitochondrial ATP production did not have an effect on either organelle trafficking (Kaasik et al., 2007), indicating that sterical hindrance by itself may already influence cellular processes critically.

Although found in almost every neurodegenerative disease, pathological aggregates are not identical in composition or dynamics between them. For instance, in FUS-FTD, we find amorphous, non-amyloidogenic aggregates. Yet a common neuropathological feature of PD, AD, and HD is the presence of an aggregation-prone disease protein that acquires amyloidogenic properties, causing it to form intracellular amyloid aggregates or extracellular amyloid plaques in the brains of patients (Sin and Nollen, 2015).

The specificity of pathological SGs to neurons might be related to their unique feature of having to constantly transport RNA granules over large distances (long axons or broad dendritic arbors), and hence contain a much higher amount of RNA granules and RBPs. This process is very advantageous, as it is much more convenient to transport small RNAs through narrow cell compartments rather than large proteins. It allows for less sterical hindrance and therefore faster transport in a viscous environment such as the cytoplasm (Wolozin and Collection, 2014). On the other hand, it also renders this system very sensitive and easily disrupted by alterations in each of the factors discussed above.

Overall, the sustained translational arrest, the toxic loss of function by trapping important regulatory proteins, and the templated misfolding of RBPs contribute greatly to the pathology of neurodegenerative diseases. Consequently, the study of membraneless structures such as SGs and their regulators and dynamics as a major hallmark in these pathologies is of great importance in order to reveal the complete disease mechanisms. In the following, we discuss several methods for the investigation of the mechanical properties and dynamics of SGs, axonal transport, and cell mechanics *in vitro* and *in vivo*.

## Selected Methods to Study Cell Mechanics

### Atomic Force Microscopy

Atomic force microscopy-based indentation measurements allow the quantification of the viscoelastic properties of biological materials (Vinckier and Semenza, 1998; Butt et al., 2005; **Figure 1B**). The key component of the indentation setup is the cantilever - a flexible spring leaf with a defined spring constant. The cantilever is equipped with a sharp tip or spherical bead that is mounted perpendicular to the cantilever axis. The indentation setup comprises furthermore a laser that is reflected from the very end of the cantilever to a four-quadrant photodiode that detects the position of the reflected laser beam, and thereby the bending of the cantilever (**Figure 1B**). An indentation measurement commences with the cantilever approaching the sample surface in a piezo-controlled fashion. Upon establishing contact, the cantilever indents the sample by a certain depth and is retracted thereafter. The interaction between the tip and the sample causes the cantilever to deflect, which results in a displacement of the laser beam on the photodiode. During this approach and retraction process, the cantilever deflection is recorded and plotted as a function of the piezo height. An indentation measurement with a calibrated cantilever yields a force-distance curve which describes the force applied to the cantilever (measured as deflection) with respect to the distance between indenter and sample. As the indenter is pushed into the sample upon establishing contact (negative distance), the force-distance curve assumes an increasingly steep slope that is commonly referred to as the indentation segment. This indentation segment is used to determine the resulting indentation depth and the Young’s modulus of the probed sample region by applying appropriate mechanical models (Hertz, 1882; Sneddon, 1965; Johnson et al., 1971; Derjaguin et al., 1975; Tabor, 1977; Johnson and Greenwood, 1997).

While the aforementioned indentation measurements are considered static and provide access to the elastic material properties, dynamic measurements allow the quantification of both elastic and viscous material properties (Mahaffy et al., 2000, 2004; Alcaraz et al., 2003). During dynamic indentation measurements, the cantilever is sinusoidally oscillating while in contact with the sample thereby applying an oscillatory stress. The sample is responding by displaying an oscillatory strain that shows a phase lag with respect to the driving force. The phase lag is used to determine the extent of viscosity in the sample, i.e., 0° phase lag indicates a purely elastic solid-like material and 90° phase lag indicates a purely viscous liquid-like material. The dynamic mode of testing allows quantifying the complex shear modulus of the probed viscoelastic material, which is calculated as the complex ratio in the frequency domain between applied stress and resultant strain (Ferry, 1980). This modulus can be used to determine the degree of solid- or liquid-like mechanical behavior (Alcaraz et al., 2003).

As both static and dynamic measurements have been performed with various cell types (Radmacher et al., 1996; Rotsch and Radmacher, 2000; Alcaraz et al., 2003; Mahaffy et al., 2004), including neurons (Lu et al., 2006), and under various conditions (Chiou et al., 2013; Rother et al., 2014), it appears likely that such measurements will also help to elucidate the material properties of impaired motor neurons. Furthermore, such indentation setups are usually equipped with inverted (fluorescence) microscopes that allow for mechanical testing and simultaneous (fluorescence) microscopy, which might provide a direct correlation between mechanical properties and organelle function in the course of neurodegenerative disease progression.

The indenter and parameter settings of the indentation setup can be chosen to specifically target small structures to determine local mechanical properties with high spatial resolution or to deform entire cells and tissue regions to probe a global viscoelasticity. Thus, AFM-based indentation measurements are capable of covering a wide range of sample dimensions with great spatial and force resolution. However, indentation testing is a surface method and cannot probe inside intact cells in order to assign distinct mechanical properties to individual intracellular structures. It requires physical contact between indenter and sample, and can therefore necessitate an elaborate sample preparation procedure, e.g., tissue dissection or cell isolation, that might introduce structural damage and therefore measurement artefacts (Schlüßler et al., 2018; Weickenmeier et al., 2018). The physical contact between indenter and sample might also provide mechanical cues to initiate intracellular structural changes that give rise to changes in mechanical properties during measurements. Such potential sources of artefacts can only be eliminated by employing contact-free methods that allow *in vivo* mechanical testing such as ODT or BM.

### Optical Diffraction Tomography and Brillouin Microscopy

Optical diffraction tomography (**Figure 1B**) is a label-free three-dimensional (3D) imaging technique, which measures the 3D refractive index distribution of transparent biological samples including tissues and cells. The 3D refractive index distribution is reconstructed by the Fourier diffraction theorem from the 2D complex optical fields measured under various incident angles (Wolf, 1969; Müller et al., 2015). The technique provides morphological and biochemical information and allows the calculation of protein concentration, dry mass, cellular volume, and sphericity of individual cells non-invasively with high spatial resolution (∼100 nm in the lateral direction) (Barer, 1952; Popescu et al., 2008; Kim et al., 2014; Yoon et al., 2015). ODT has been used on neurons to study the dynamical behavior of living dendritic spines (Cotte et al., 2013) and morphological changes in early neurodegenerative progress in PD neurons (Yang et al., 2017). Time-lapse measurements of dry mass of individual cells and tissues from 2D quantitative phase microscopy (QPM) reveal dynamics of the specimens, investigating the growth rate and mass transport in neuronal networks (Cintora et al., 2017). Furthermore, temporal correlation of the series of time-lapse 2D phase maps provides quantitative analysis of intracellular diffusion and directed motion in glia and hippocampal neurons (Wang et al., 2011) and *Drosophila* oocytes (Drechsler et al., 2017). The principle of the temporal correlation analysis in 2D QPM can be extended to the time-lapse ODT measurement due to the fast tomogram acquisition rate of ODT (∼10 tomograms/s) (Kim et al., 2013), which can detect region-specific dynamics and viscoelastic properties in motor neurons in 3D.

Confocal BM (**Figure 1B**) allows the measurement of the viscoelastic properties of tissues and single cells *in vivo*. It is based on an inelastic scattering process, the so-called Brillouin scattering, between the incident light and periodic mass density fluctuations due to travelling sound waves inherent to the sample. The scattering results in a frequency shift depending on the longitudinal modulus, the density and the refractive index of the sample (Dil, 1982). BM achieves diffraction-limited resolution similar to confocal fluorescence microscopy, requires no labeling of the specimen and, in combination with ODT, allows the calculation of a 3D map of the longitudinal modulus of the sample. The technique has been used successfully to measure mechanical properties of human cornea (Scarcelli et al., 2011, 2014; Scarcelli and Yun, 2012), ruminant retina (Courrol et al., 2007; Weber et al., 2017), murine carotid arteries (Antonacci et al., 2015), rabbit bone tissue (Fukui, 2011), zebrafish embryos (Fujimura et al., 2007; Meng et al., 2016), zebrafish larvae (Schlüßler et al., 2018) as well as single fibroblasts (Scarcelli et al., 2015). BM and ODT are also be the perfect pair to quantitatively study the physical properties of phase-separated, membraneless compartments, and in particular the liquid-to-solid transitions of SGs inside living neurons. Together, these various techniques might be used to study all mechanical changes in neurodegenerative diseases as discussed above.

## Conclusion

In the past years, knowledge about neurodegenerative diseases has enormously increased, but the underlying disease mechanisms still remain unknown. Since transport deficiencies seen in neurodegenerative diseases are caused by an impaired cytoskeleton, this is likely to affect the mechanical properties of the cell. Also, aggregate formation and phase transition processes can contribute to local mechanical property changes (Hyman et al., 2014). Diffusion and active processes required for axonal transport could be impaired due to the liquid-to-solid phase transition of axonal proteins in the aforementioned pathological conditions.

Using mechanical and optical measurement techniques, the assessment of the mechanical properties of neurodegenerative disease models (e.g., rodent and cell culture) presents a novel approach to investigate their underlying mechanisms. These techniques are especially well suited to explore the properties of tissues, single cells as well as subcellular compartments. Hence, global mechanical changes generated by the cytoskeleton and associated proteins can be detected and quantified. While AFM is a well-established technique to measure mechanical material properties, it requires direct physical contact between probe and sample. This could potentially trigger active cytoskeleton change inside the sample during measurements and thereby introduce artefacts. Optical methods such as BM and ODT can be used to access mechanical properties non-invasively which can be particularly interesting for phase separation processes and protein aggregate formation as seen in models for neurodegenerative diseases. By combining the presented techniques with methods that allow the determination of structural characteristics, a comprehensive picture that links structure and function will emerge.

It can now be investigated how the mechanical properties change during the course of neurodegeneration and which neuronal components are affected most (e.g., proximal or distal axon, growth cones, soma, etc.). Moreover, early cytoskeletal damage might be reflected by mechanical properties which can serve as an initial marker for neuronal degeneration and death. It is also possible that certain structural changes occur explicitly in order to change the mechanical properties for the benefit of the cell, e.g., to stabilize the cell and keep transport processes running. Cell mechanics, since always coupled to biochemical and physiological processes, can provide new insight into the basic understanding of neurodegenerative diseases and can help to identify mechanism involved in the emergence of such disorders.

## Author Contributions

MN, GR, and SM wrote the first draft of the manuscript. AS, RS, and KK wrote sections of the manuscript. All authors contributed to the manuscript revision, and read and approved the submitted version.

## Conflict of Interest Statement

The authors declare that the research was conducted in the absence of any commercial or financial relationships that could be construed as a potential conflict of interest.
